# Psychosocial Factors Affecting Parental Report of Symptoms in Children: A Systematic Review

**DOI:** 10.1097/PSY.0000000000000767

**Published:** 2020-02-07

**Authors:** Louise E. Smith, John Weinman, Jenny Yiend, James Rubin

**Affiliations:** From the Institute of Psychiatry, Psychology and Neuroscience (Smith, Yiend, Rubin); and School of Cancer and Pharmaceutical Sciences (Weinman), King’s College London, London, United Kingdom.

**Keywords:** symptoms, child symptoms, psychological factors, parents, **ADHD** = attention-deficit/hyperactivity disorder, **SDQ** = Strengths and Difficulties Questionnaire

## Abstract

**Objective:**

Parents make important treatment decisions for their children based on symptoms they perceive their child to be experiencing. Multiple psychological factors are associated with subjective symptom perception, but factors affecting perception of symptoms in others have been explored less. We systematically reviewed the literature to identify parent and child psychological factors associated with parental report of physical symptoms in their child.

**Methods:**

We searched Embase, Ovid, PsycINFO, and Scopus for studies that investigated associations between psychological factors and parental report of symptoms in their child.

**Results:**

Thirty-six citations reporting on 34 studies that assessed the association between parent or child psychological factors and parental report of physical symptoms in the child were included in the review. Three main factors were identified as being associated with parental symptom report. First, there was evidence for an association between parental symptom report and affect, in particular parent and child anxiety. Second, child behavioral and conduct problems, and temperament-related challenges (problems with feeding and sleeping) were associated with parental symptom report. Third, parental expectations and beliefs that symptoms would occur were associated with parental symptom report, although few studies investigated these associations.

**Conclusions:**

Parent and child affect, and parental expectations and beliefs may influence parents’ cognition, causing them to pay more attention to their child, interpret their child’s behavior as symptomatic, and recall symptoms in the child. Given the importance of parental perception of symptoms in driving decisions around care, additional research in this field is needed.

## Abstract 2

Supplemental digital content is available in the text.

## INTRODUCTION

Symptom perception is a complex process. Although a clear correspondence between pathology and symptom occurrence and severity was once presumed, there is now convincing evidence that psychological factors, such as the wider context, the behavior of others, and the attitudes of the person involved, can influence whether one perceives a symptom (1). Models of subjective symptom perception postulate that bodily sensations lead to symptom experience through cognitive processing such as attention to the bodily sensation and interpretation of the sensation as a symptom (see Van den Bergh et al. (2) for summary). Psychological factors such as trait negativity, health anxiety, and learning are proposed to moderate these processes. However, relatively little research has investigated factors affecting the perception of symptoms in someone other than oneself, such as a child or dependent.

The ability to accurately identify symptoms in others is particularly important for parents. If parents are unable to accurately perceive symptoms in their child, they might incorrectly detect or miss signs of illness, symptoms of allergy or intolerance, or adverse effects of medications, and make inappropriate decisions for their child regarding medical care, life-style, or medication adherence as a result. Perceived food intolerance is one example of this. Approximately one-third of parents believe that their child has food sensitivity (3). However, most of these children do not undergo any formal testing of food allergy such as skin prick tests or oral food challenges. When formal testing does occur, the actual prevalence of food hypersensitivity is much lower (approximately 1.9%–4.5%) (3,4).

Although formal data are scarce, one study based on parental report estimated that 56% of children aged 3 to 5 years have experienced symptoms such as headache, stomach ache, tiredness, and dizziness in the last 14 days (5), a broadly similar rate to that seen in adults (6–8). However, agreement between parent-reported and child self-reported symptoms is varied. For example, one study found that, although parent-child agreement was relatively high for headache frequency, agreement was lower for other pain symptoms (9). Another study reported similar results, finding that parent and child symptom reports were highly correlated in children with recurrent stomach aches, but were less strongly correlated in well children and not correlated in children with emotional disorders (10). These results suggest that the process of parental perception of symptoms in one’s child is not straightforward and may depend in part on the type of symptom observed.

Multiple psychological factors have been identified as relevant in subjective symptom perception. In particular, heightened symptom expectations (11,12), psychological traits such as anxiety (13), depression (14), and negative affect (15), as well as beliefs relating to exposures that might trigger symptoms (16), have been associated with symptom reporting. However, it is unclear if the predictors of symptom perception in oneself are the same as those for perceiving symptoms in one’s child. While perception of symptoms in oneself is driven by detection of internal cues and bodily sensations, parental perception of symptoms in one’s child relies on external cues, such as observations of the child’s behavior, or listening to and assessing self-reports from the child. Parents of young or severely disabled children who are unable to verbalize their bodily sensations may have to rely solely on observation of the child’s behavior.

Psychological factors known to be influential in subjective symptom perception may also affect parents’ perception of symptoms in their child. Parents with higher trait negativity may pay more attention to their child’s behavior, be more likely to interpret their child’s behavior as symptomatic, and recall symptoms perceived in the child more readily or frequently (2). Parental expectations for symptoms to develop and beliefs about symptoms may influence these cognitive processes. The child’s behavior, affecting how they display symptoms experienced, may also influence parental symptom perception. It is likely that all three factors are important and interact with each other. To identify parent and child psychological factors that are associated with parent-report of physical symptoms in one’s child, we conducted a systematic review of the available literature. We used search terms relating to parents, perception of symptoms, and symptoms that children might commonly experience.

## METHODS

We conducted a systematic review in accordance with PRISMA criteria (17) to identify factors associated with parental perception of symptoms in children. We searched Embase, Ovid, and PsycINFO through OvidSP, and Scopus. The final search used the terms (Parent* ADJ3 (perception OR perceive)) AND (side effect OR symptom* OR pain* OR asthma*). Asthma was included in the search terms because it is a condition experienced commonly in childhood, which was prevalent in our preliminary searches. Medical Subject Headings terms were also searched where possible. Databases were searched from inception to July 12, 2018. References and forward citations of included articles were also searched.

### Inclusion Criteria

The following inclusion criteria were used:

Participants: Studies were included if they investigated parents of children aged 0 to 18 years. Studies were excluded if parents discussed symptom report outcome measures with their child or if it was unclear whether the parent or the child completed outcome measures.
Predictors/Exposures: Studies were included if they investigated the association between psychological or social factors and parental report of symptoms.
Outcomes: Studies were included if the outcome was parental report of physical symptoms in the child, including pain, asthmatic symptoms, adverse effects from medication, or perceived allergy or food intolerance. Outcomes relating to parental contact with health professionals after symptom perception were excluded. Outcomes based on parental report of a diagnosis for the child by a health care practitioner were also excluded.
Study reporting: Only studies published in English were included.

### Data Extraction

We extracted information on study design, inclusion criteria, number of participants, child age, symptom type, symptom measure used, and predictors of symptom report.

### Risk of Bias

Risk of bias was measured using an amended version of the Downs and Black checklist (18), as in previous systematic reviews (19). The Downs and Black checklist assesses the methodological quality of randomized and non-randomized studies (18). This version did not include items referring to interventions because they were not relevant for any included study. The Downs and Black checklist has been validated (20) and is suitable for use in systematic reviews (21). Five aspects of the studies’ methods were assessed: reporting (out of 10), internal validity (bias; out of 3), confounding (selection bias; out of 3), external validity (out of 2), and statistical power (1 item). Scores were added to give a total of up to 19. We rated studies as good quality if they scored 16 or over, moderate quality if they scored 11 to 15, and poor quality if they scored 10 or less. Studies scored poorly for reporting if they scored 6 or lower; internal validity (bias), confounding (selection bias) and external validity if they scored 1 or lower; and if they did not include a justification for the sample size used.

### Procedure

L.E.S. came up with the search terms, carried out the search, screened articles, extracted data, and completed risk of bias assessment with guidance from J.R. All authors helped devise the idea for the review, assisted with interpretation of results, and critically reviewed and revised the manuscript. Factors were grouped according to psychosocial factor.

## RESULTS

### Study Characteristics

**FIGURE 1:**
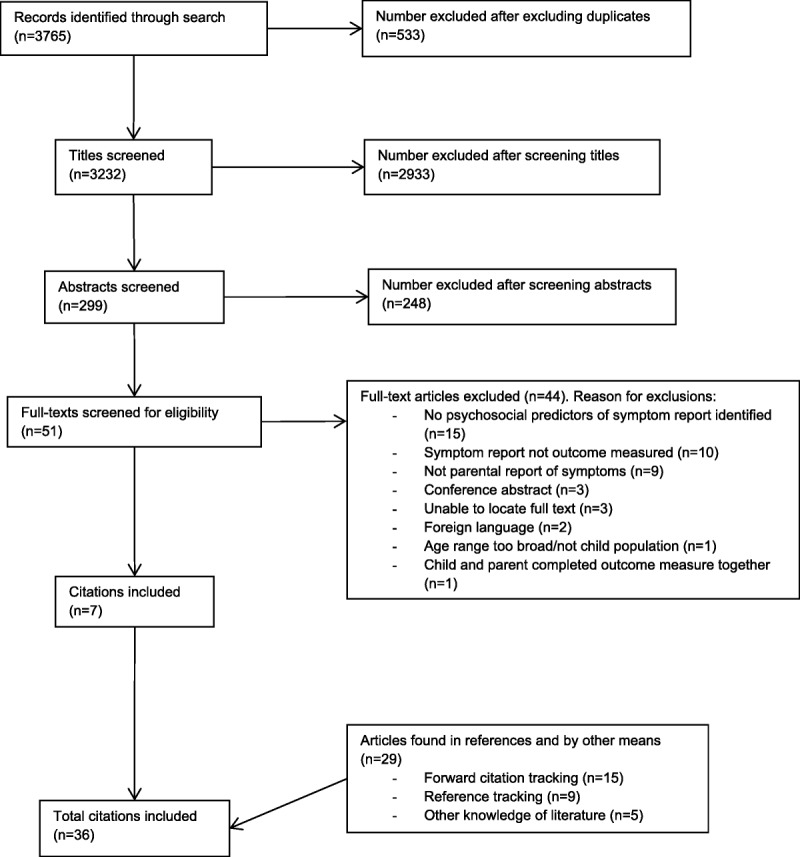
Flowchart depicting the selection of studies included in the systematic review with reasons for exclusion.

A total of 3765 citations were found by the original search. After removing duplicates, 3232 citations remained. After title, abstract, and full-text screening, seven citations remained. Reference searching and forward citation tracking identified a further 29 citations that met the inclusion criteria, giving a total of 36 citations reporting on 34 studies (Figure 1). Twenty-three studies used a cross-sectional design, eight used a cohort design, and three used case-control design (Table 1). Nine studies investigated somatic symptoms in general, with a further nine investigating solely headache, three investigating stomach ache, and two investigating both headache and stomach ache; one investigated recurrent symptoms (Supplemental Digital Content, http://links.lww.com/PSYMED/A603). Six studies investigated pain. Two studies investigated the incidence of symptoms in response to vaccination, one investigated symptoms attributed to food allergy, and one investigated symptoms attributed by parents to various ailments such as the common cold.

**TABLE 1:**
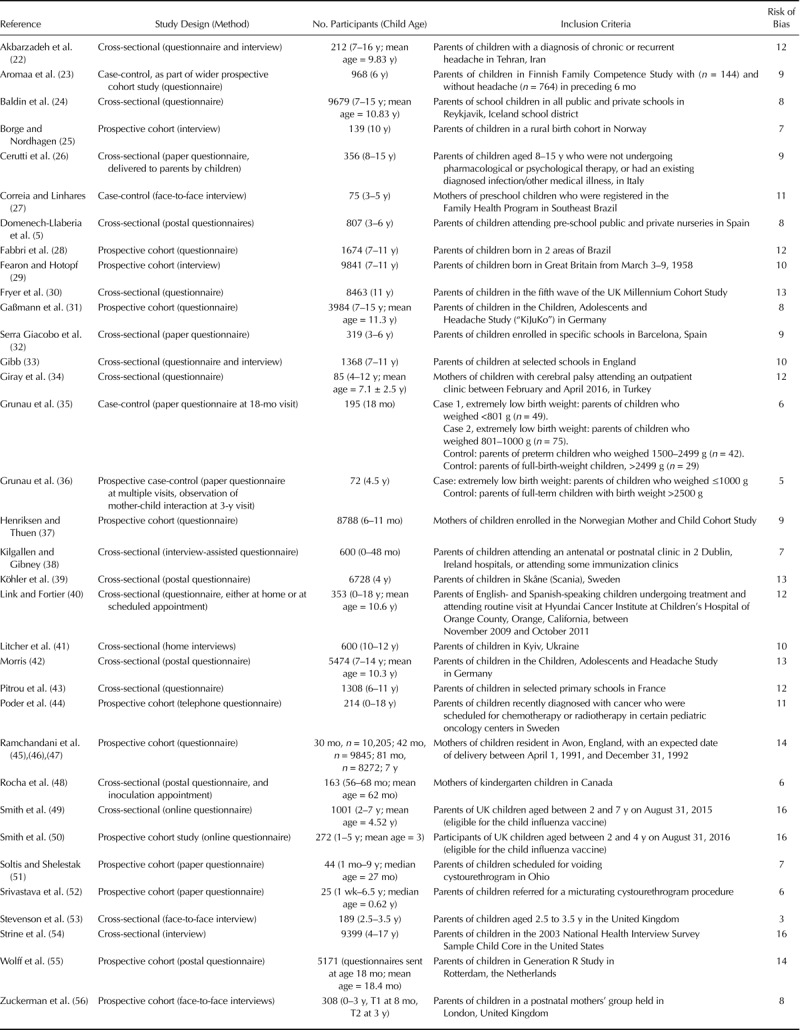
Methods of Articles Included in Systematic Review

### Risk of Bias

**FIGURE 2:**
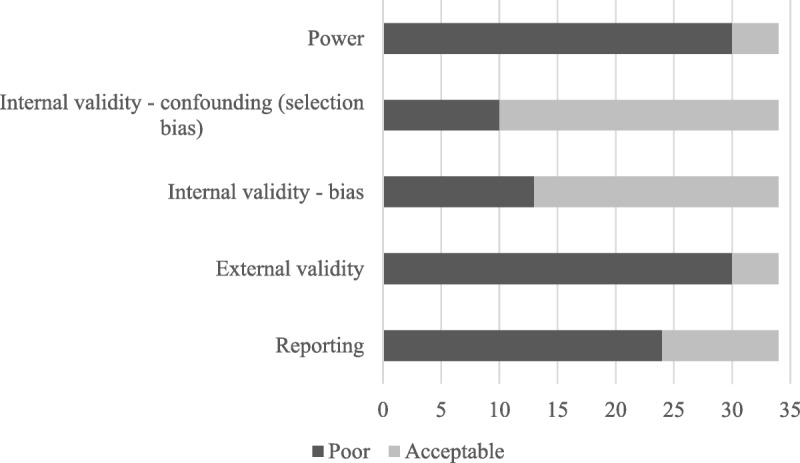
Chart indicating number of studies displaying different aspects of risk of bias.

Scores ranged between 3 and 16 of a possible 19 (Table 1). Most studies were poor quality (*n* = 19), with 12 moderate-quality studies. There were three good quality studies. Only four studies gave a justification for the sample size used (28,42,49,50) (Figure 2). With respect to internal validity, 10 studies scored poorly for confounding (26,32,33,35,36,41,48,51–53) and 13 scored poorly for bias (5,23,25,26,32,35,36,38,41,48,52,53,56). External validity was acceptable in only four studies (30,38,49,54). Reporting was poor in 24 studies (5,22–38,44,48,51–53,56).

Only studies that were moderate or good quality are reported narratively.

### Instruments Used to Measure Parental Perception of Symptoms

Studies used many different measures of parental perception of symptoms. Few studies used validated questionnaires to measure parental perception of symptoms. Three studies used the parent-report version of the Children’s Somatization Inventory (57), with another study using the short version; one study used the Child Behaviour Checklist (58); one study used a modified version of the Memorial Symptom Assessment Scale (59); and another used the Non-Communicating Children’s Pain Checklist – Revised (60). All other studies used their own measure of parental perception of symptoms; this was often a single item asking how frequently the child had experienced a given symptom over a certain period (see supplementary materials for full details, http://links.lww.com/PSYMED/A603).

### Predictors of Parental Symptom Report

Parent and child psychosocial factors associated with parental report of symptoms are reported in Table 2. Where studies used adjusted analyses, only these are reported. Many studies used the Strengths and Difficulties Questionnaire (61), which is made up of five components: emotional problems, conduct problems, hyperactivity-inattention, peer problems, and prosocial behavior. Where possible, we have reported each component individually.

**TABLE 2:**
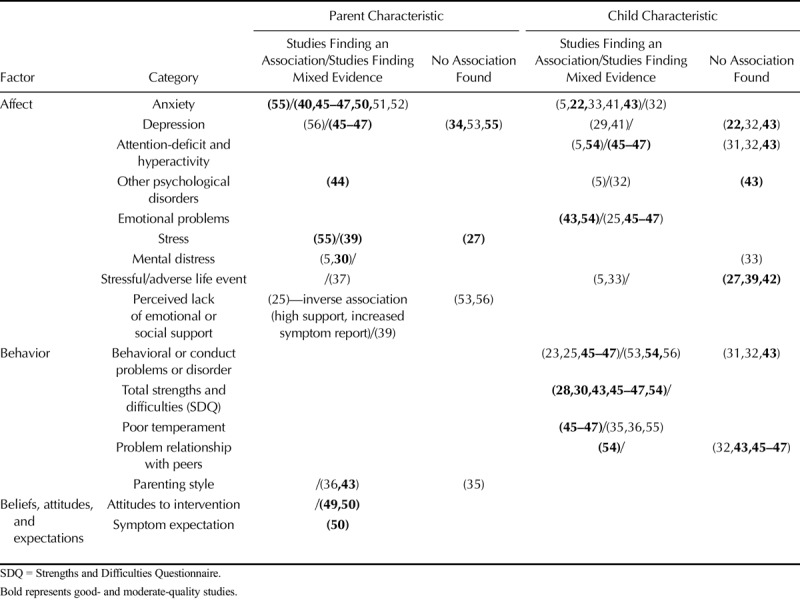
Summary of Studies Investigating the Association Between Psychosocial Characteristics and Parental Report of Physical Symptoms in the Child

#### Parent Psychosocial Characteristics

There was some evidence for associations between parental affect and parental symptom report. Evidence was stronger for the role of parental anxiety than other psychological traits or stressful events. Studies measured trait anxiety, apart from two which investigated anticipatory and experienced anxiety (51,52), and another which used the Crown-Crisp index (45–47). One study found an association between parent anxiety and report of somatic symptoms (55), whereas three studies found mixed evidence of an association with increased report of child chronic pain, recurrent stomach aches, and perception of adverse effects from influenza (40,45–47,50). One study found evidence for an association between maternal depression and reporting of recurrent child symptoms (45–47), whereas two studies (34,55) found no association between parental depression and presence and frequency of parent-reported somatic complaints and child pain. Parental post-traumatic stress disorder was associated with symptom report (44). There was mixed evidence for an association between parent distress or stress and parental symptom report. Distress was associated with frequency of parent-reported pain in one study (30). Three studies investigated the association between parental stress and parent-reported symptoms. One investigating somatic symptoms found an association (55); one investigating recurrent stomach aches found mixed evidence for an association (39); and one investigating general physical symptoms found no association (27). Paternal, but not maternal, low emotional support was associated with report of recurrent stomach aches (39).

Few studies investigated the association between parental behavioral factors and parental symptom report. One study found an association between punitive behaviors and less frequent report of headaches (43).

There was some evidence for an association between negative parental beliefs and attitudes and parental symptom report. Two studies investigated the association between parental reporting of adverse effects from the child influenza vaccine and multiple parental beliefs, such as believing that vaccines cause adverse effects, and attitudes, such as not liking vaccines in general (49,50). Beliefs and attitudes were associated with parental report of adverse effects in both studies (see supplementary materials for full details, http://links.lww.com/PSYMED/A603). One of these studies also investigated parents’ expectation that their child would experience a symptom, finding an association between parental expectation of adverse effects and later adverse effect reporting (50).

#### Child Psychosocial Characteristics

There was mixed evidence for associations between child affect and parental symptom report. Evidence was strongest for an association between parental symptom report and child anxiety, emotional problems, and attention-deficit/hyperactivity disorder (ADHD). Child emotional problems were associated with parent-reported frequent or severe headaches in two studies (43,54), whereas another study found mixed evidence for an association with parent-report of recurrent symptoms (45–47). Studies investigating child anxiety used measures that could be used as diagnostic tools for general anxiety disorder. Increased parent-reported presence, frequency, and severity of headaches were associated with child anxiety in two studies (22,43). One study investigated parent-reported child anxiety (22), whereas the other investigated child-reported anxiety (43). One study found an association between ADHD and frequency and severity of parent-reported headache (54), whereas another found mixed evidence for an association with recurrent stomach ache (45–47). One study found no evidence for an association between ADHD and frequency of parent-reported headache (43). Two studies found no association between child depression (parent-reported child depression (22), child-reported depression (43)) and presence, frequency, and severity of parent-reported headache. Parent-reported anxiety and depression were not associated with parental report of recurrent headache (42). Three studies found no association between adverse or stressful life events and parent-reported headache or stomach ache (27,39,42). There was also no evidence for an association between oppositional defiant disorder, social phobia, or separation anxiety and parent-reported frequent headache (43).

There was some evidence for an association between child behavioral factors and parental symptom report. All studies that investigated whether total high difficulties on the Strengths and Difficulties Questionnaire were associated with parental symptom report found evidence for an association (28,30,43,45–47,54). Conduct problems were associated with parent-reported recurrent stomach ache in one study (45–47), whereas another investigating parent-reported headaches found mixed evidence (54). Temperament-related challenges, such as problems with feeding or sleeping, were also associated with parent-reported recurrent stomach ache (45–47). Evidence for an association between child problematic relationships with peers and parental symptom report was mixed, with one study investigating parent-reported headaches finding an association (54); two further studies investigating parent-reported headache and recurrent abdominal pain found no association (43,45–47).

## DISCUSSION

Although mechanisms underlying symptom perception in oneself are more clearly understood, less research has explored psychological factors associated with parental perception and report of symptoms in one’s child. Our review identified three broad categories of factors affecting parental report of symptoms: affect, behavior, and expectations and beliefs about symptoms. These build upon categories previously identified in the literature (62).

There was good evidence for an association between parental anxiety and report of symptoms in the child, but less evidence for associations with other psychological traits. In models of subjective symptom perception (e.g., Ref. (2)), anxiety is thought to heighten attention to bodily sensations and lower the threshold at which sensations are detected (13,63). In parental perception of symptoms in one’s child, heightened parental anxiety may increase attention to the child’s behavior and may cause a more negative interpretation of the reasons underlying ambiguous behaviors. There was also evidence that child anxiety, as well as emotional problems and ADHD, were associated with parental report of physical symptoms. Because somatic symptoms are common in children with anxiety (64) and other emotional and behavioral disorders (65), this finding is perhaps unsurprising.

Most of the research investigating the association between behavioral factors and parental symptom report has focused on child, rather than parent, behavioral factors. How child behavioral difficulties, such as having problems with peers, may affect parental symptom report is poorly understood. Although all studies investigating child temperament in the review found an association with parent-reported symptoms, no rationale was given for investigating these factors. One mechanism that may explain this pattern of findings is that children perceived as “difficult” may verbally report more symptoms, leading to increased parental symptom report. In addition, parents may pay more attention to their child’s behavior if he/she are perceived as “difficult”, causing parents to notice and report more symptoms. Children may also behave differently in the presence of their parents, leading to increased possibility of symptom detection by parents. For example, children display more pain in the presence of a parent than a stranger (66). Better quality research is necessary to clarify the nature of, and reasons underlying, associations between child behavioral factors and parental symptom report.

The effects of some psychosocial factors on parental symptom report were conspicuous by their absence in our review. In particular, only one study investigated the effect of parental expectation of symptoms (50). Given the wealth of evidence suggesting that expectation influences symptom perception in oneself (11,12,67,68), it is surprising that so few studies have investigated the influence of parental expectation on parent-reported symptoms. One possible explanation for this dearth of research is that studies have so far focused on finding factors associated with increases in symptoms experienced by the child rather than parental report of symptoms. However, given that decision making about medical treatments or potential life-style adjustments will be made based on parental perception and recall of symptoms, it is important to identify factors that may influence this process.

### Limitations of Studies Included in the Review

Most studies included in our review were poor quality. In particular, studies fell short on reporting and external validity. Studies used a wide range of questions to assess parental report of symptoms in the child, with very few studies using validated measures. Studies were also heterogeneous with regard to their populations, investigating parents of children of different ages. Statistical analyses were also heterogeneous, with some studies using inappropriate tests, not reporting statistical tests used (53,56), or using small sample sizes. Symptom report was also defined differently by different studies, with some studies using higher thresholds for symptom report than others. Studies included in the review used many different scales to measure the same construct; this was particularly notable for temperament and behavior (69) and made it difficult to compare results between studies.

### Limitations of the Review

Several limitations of our review should also be considered. First, symptoms perceived by parents in this review were wide ranging. We were unable to investigate whether predictive factors differed in relation to different symptoms. However, literature investigating the nocebo effect supports the notion that factors such as expectations and anxiety are associated with perceiving a wide range of subjective symptoms (11).

Second, we did not differentiate predictors of parent-report of child symptoms by age of the child. This was due to the wide age range used in some studies (e.g., 0–18 years) and the small number of studies investigating each factor. Although it is likely that some child psychological factors, such as school-based stressors, would be more prevalent in older rather than younger children, no studies included in the review investigated these. The lack of comparable measures for psychosocial factors, such as temperament, across age groups is recognized as a problem for the identification, and relative importance, of factors associated with medically unexplained symptoms in children and adolescents (70).

Third, few studies investigated the same factors, meaning that our conclusions for some risk factors are based on limited results and should be treated with caution.

Fourth, it was notable that only seven citations were identified through our search strategy, with most citations being found through forward citation and reference tracking. To date, parental perception of child symptoms has rarely been studied as a topic in its own right and has no specific easily searchable terminology, making relevant data difficult to find. It is likely that other studies investigating relevant risk factors exist, but we were unable to locate them.

Fifth, we restricted our search to psychosocial predictors of parental symptom report. Other studies exist investigating personal and clinical factors such as breastfeeding (71), smoke exposure (72), exposure to indoor dampness and mold (73), attending day care (74), and number of siblings (75), particularly in relation to child asthma and allergic symptoms. A full model of parental symptom perception may need to account for these factors.

Sixth, we used parental report of symptoms as a proxy for parental perception of symptoms. Retrospective symptom reports are often biased compared with momentary symptom assessments, with the former often leading to greater estimates of symptoms (76). This is likely due to multiple memory biases playing a role in retrospective reports [see Van den Bergh and Walentynowicz (76) for a review]. Because diary methodologies were not used by any of the studies included in the review, parental report of symptoms may have been affected by these factors and therefore may not have mapped exactly onto symptom perception. However, because retrospective parental reports of symptoms are commonly relied upon by physicians when making diagnoses for children and by parents when making health-related decisions for their child, it is important to identify psychological factors that may influence parental report of symptoms in the child.

Lastly, an important question to consider is whether parent and child psychosocial factors are associated with increased symptoms experienced by the child or increased parental detection of symptoms, irrespective of the child’s subjective experience. We were unable to differentiate between these outcomes. This distinction has already been identified as a concern in the literature (55), and it is likely that both mechanisms are relevant (40,45,46,55).

## CONCLUSIONS

Psychological factors from three categories were found to be associated with parental report of symptoms: affect; behavior; and expectations, attitudes, and beliefs. The influence of both parent and child affect was investigated. Factors most often associated with parental report of symptoms were parent anxiety and stress, and child anxiety, emotional problems, and ADHD. Behavioral factors were mostly investigated with reference to the child, with problems in conduct and temperament being consistently associated with greater parental report of symptoms. Beliefs, attitudes, and symptom expectations may also influence parent symptom report, but there was a dearth of research investigating these factors. Better quality research using more standardized methods and measures is needed to more fully understand the impact of, and mechanisms through which, psychosocial factors influence parental report of symptoms.
